# Dramatic clearance of periorbital lupus miliaris disseminatus faciei with tapinarof 1% cream

**DOI:** 10.1016/j.jdcr.2025.10.054

**Published:** 2025-11-05

**Authors:** Jun Omatsu, Noboru Oshima

**Affiliations:** a Department of Dermatology, University of Tokyo Graduate School of Medicine, Bunkyo-ku, Tokyo, Japan; b Shibuya Ekimae Oshima Dermatology Clinic, Tokyo, Japan

**Keywords:** aryl hydrocarbon receptor, granulomatous dermatoses, lupus miliaris disseminatus faciei, tapinarof

## Introduction

Lupus miliaris disseminatus faciei (LMDF) presents as perifollicular granulomas with central caseous necrosis in the periorbital region and typically follows a stubborn, scarring course.^1^

Current treatments—including tetracyclines, systemic steroids, cyclosporine, and isotretinoin—often give only partial or short-lived responses.^2^

Tapinarof 1% cream is a first-in-class, non-steroidal aryl-hydrocarbon-receptor (AhR) agonist that received U.S. FDA approval in 2022 for adult plaque psoriasis and in 2024 for atopic dermatitis.^3^

Beyond its Th17-suppressive and skin-barrier-restorative activities, tapinarof directly dampens LL-37-induced mast-cell degranulation, down-regulating chymase-1, tryptase-β, MMP-9, TNF-α and IL-6—mediators implicated in granuloma formation.^4^

A recent report documenting rapid clearance of refractory granulomatous rosacea with topical tapinarof^5^ prompted us to explore its use in recalcitrant LMDF.

## Case report

**Fig 1:**
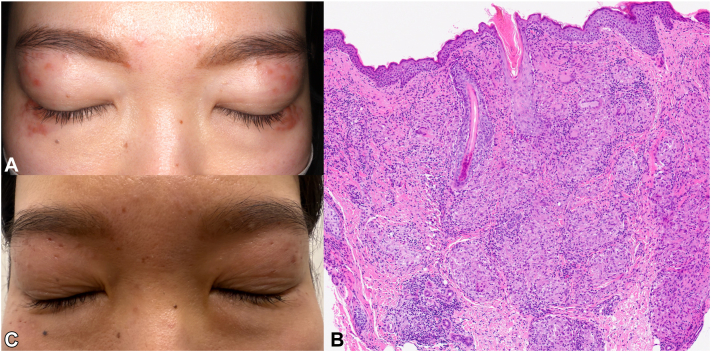
**A,** Baseline periorbital LMDF showing multiple 2 to 4 mm red-brown papules and nodules on upper and lower eyelids. **B,** Periorbital skin biopsy showing perifollicular epithelioid granulomas with central caseous necrosis and scattered multinucleated giant cells (hematoxylin–eosin, ×100). **C,** Six weeks after nightly tapinarof 1% cream: inflammatory lesions cleared; faint erythema, and shallow pits remain.

A 32-year-old Japanese woman presented with a 3-year history of asymptomatic, 2-4 mm red-brown papules and nodules clustered on her upper and lower eyelids (Fig 1, *A*). She denied photosensitivity, systemic symptoms or tuberculosis exposure. Skin biopsy showed perifollicular epithelioid granulomas with central caseous necrosis and multinucleated giant cells; Ziehl–Neelsen and periodic-acid–Schiff stains were negative. Based on the characteristic histopathologic findings and exclusion of sarcoidosis, lupus vulgaris, and granulomatous rosacea, the diagnosis of LMDF was confirmed.

Oral prednisolone 20 mg/day for 8 weeks flattened some lesions, but tapering below 10 mg/day caused rebound. Over the next 18 months she failed cyclosporine (100 mg/day, 8 weeks), pentoxifylline (400 mg 3 times daily, 4 weeks), minocycline (100 mg/day, 12 weeks), doxycycline (200 mg/day, 12 weeks) and topical ivermectin 1% and metronidazole 0.75% gels. After discontinuing all prior therapies, tapinarof 1% cream (1 fingertip unit per periorbital area) was initiated nightly after a 2-week washout period.

By week 2, lesions had flattened; by week 6, inflammatory papules had resolved, leaving faint erythema and a few shallow 1 to 2 mm pits (Fig 1, *C*). Tapinarof was discontinued at week 8. Ten weeks later, 3 new papules appeared on the right upper eyelid; tapinarof was re-started, achieving clearance within 14 days. A thrice-weekly maintenance regimen has since maintained remission. No adverse effects were reported.

After 6 months of follow-up, the patient remains lesion-free. The shallow pits noted at week 6 have progressively filled in and are now barely perceptible, leaving only subtle post-inflammatory erythema. The brisk response of this patient suggests that AhR may be able to abbreviate LMDF activity as well as contribute to scar remodeling.

## Discussion

LMDF has no established therapy and often regresses only after several years, typically leaving cosmetically distressing pits.^1^ Differential diagnoses include granulomatous rosacea, sarcoidosis, and lupus vulgaris, which can present with facial papules and granulomatous inflammation. Histopathologically, LMDF is characterized by perifollicular granulomas with central caseous necrosis and absence of detectable microorganisms, distinguishing it from these mimickers. Conventional therapies such as tetracyclines, isotretinoin, corticosteroids, and immunosuppressive agents often result in partial or transient improvement, and recurrence is common after dose tapering.

Our patient’s disease persisted for 3 years despite systemic corticosteroids, cyclosporine, pentoxifylline, 2 tetracyclines, and multiple topical agents. Once-daily tapinarof 1% cream produced rapid quiescence within 6 weeks. Relapse after cessation, followed by prompt re-clearance on re-challenge and durable control on thrice-weekly maintenance, strongly implicates tapinarof as the key therapeutic factor.

Mechanistically, AhR activation down-regulates the IL-17/IL-23 axis and other Th1/Th17 cytokines sustaining granulomatous inflammation.^6^^,^^7^ AhR signalling also restores filaggrin, loricrin and involucrin expression, strengthening the epidermal barrier and reducing oxidative stress.^7^ In-vitro data further show that tapinarof attenuates LL-37–driven mast-cell degranulation and decreases MMP-9, TNF-α, and IL-6, all implicated in granuloma formation and dermal remodeling.^4^

Clinical experience with tapinarof in granulomatous disease is emerging. Complete clearance of refractory granulomatous rosacea^5^ and successful treatment of granuloma annulare^8^ support a class effect. Our case extends these observations to LMDF, suggesting that cutaneous granulomatous disorders sharing similar inflammatory circuits may be amenable to AhR modulation.

None of the common adverse effects—application-site burning or headache—reported in large tapinarof trials were observed, underscoring its favorable tolerability profile.^6^ The pits visible at week 6 gradually softened and became barely noticeable, indicating that early suppression of inflammation may also improve nascent textural change. Limitations include the single-patient design and the possibility of spontaneous remission, although the on–off response makes this unlikely.

We report a case where tapinarof appeared to be an effective and well-tolerated topical option for recalcitrant periorbital LMDF. Further studies are warranted to clarify the long-term efficacy and mechanisms of AhR agonists and to explore their potential role in LMDF and other granulomatous dermatoses.

## Conflicts of interest

None disclosed.
